# High-throughput GPU layered decoder of quasi-cyclic multi-edge type low density parity check codes in continuous-variable quantum key distribution systems

**DOI:** 10.1038/s41598-020-71534-5

**Published:** 2020-09-03

**Authors:** Yang Li, Xiaofang Zhang, Yong Li, Bingjie Xu, Li Ma, Jie Yang, Wei Huang

**Affiliations:** 1 Science and Technology on Security Communication Laboratory, Institute of Southwestern Communication, Chengdu, 610041 China; 2 School of Communication and Information Engineering, Chongqing University of Posts and Telecommunications, Chongqing, 400065 China; 3 College of Computer Science, Chongqing University, Chongqing, 400044 China

**Keywords:** Information theory and computation, Quantum physics, Information technology

## Abstract

The decoding throughput during post-processing is one of the major bottlenecks that occur in a continuous-variable quantum key distribution (CV-QKD) system. In this paper, we propose a layered decoder to decode quasi-cyclic multi-edge type LDPC (QC-MET-LDPC) codes using a graphics processing unit (GPU) in continuous-variable quantum key distribution (CV-QKD) systems. As described herein, we optimize the storage methods related to the parity check matrix, merge the sub-matrices which are unrelated, and decode multiple codewords in parallel on the GPU. Simulation results demonstrate that the average decoding speed of LDPC codes with three typical code rates, i.e., 0.1, 0.05 and 0.02, is up to 64.11 Mbits/s, 48.65 Mbits/s and 39.51 Mbits/s, respectively, when decoding 128 codewords of length ${10}^{{6}}$ simultaneously without early termination.

## Introduction

Modern computer systems place a high importance on security when it comes to sharing and transmitting data between client devices (e.g., remote participants) over computing networks. Current methods of ensuring the secret communication between clients and/or servers include implementing encryption techniques. However, encryption techniques that use shared keys, which is established with algorithms based on the assumptions of computation complexity, may no longer guarantee data security, especially with the introduction and availability of large-scale universal quantum computers.

Quantum key distribution (QKD), which establishes a secret key between two remote participants based on quantum mechanics principles, can provide the guaranteed security between the two participants by using a one-time-pad encryption algorithm to encrypt and decrypt data^1,2^. QKD has been developing rapidly in both theory and experiment since the groundbreaking work of Bennett and Brassard^1^ and so far has become one of the most mature branches of quantum information technologies.

Currently, there exist two categories of QKD. One category is the discrete variable QKD (DV-QKD), where the key information is encoded on discrete Hilbert space, and the other is the continuous variable QKD (CV-QKD), where the key information is encoded on continuous Hilbert space, such as the quadratures of coherent states. Since CV-QKD can directly utilize the standard telecommunication technologies (such as coherent detection), it has more potential advantages in practice and much progress has been made recent years^3–12^.

Generally, the two participants in a CV-QKD system desire to establish a secret key for one another over a long distance with a very low signal-to-noise ratio. Then it naturally brings a problem on how to design codes with excellent error-correction capability, under such a stringent channel condition. In this case, only low-rate codes with very long block lengths can be exploited to achieve high efficiency key reconciliation.

Low density parity check (LDPC) codes have been shown to possess Shannon-limit approaching error-correction performance^13^ and they have also been broadly applied in various communication systems, such as the DVB-S2 standard and the Enhanced Mobile Broadband (eMBB) data channels for 5G New Radio^14,15^. In Ref.^16^, the authors designed an irregular LDPC code of length ${10}^{{7}}$ which achieves within 0.04 dB of the Shannon limit. Consequently, LDPC codes with long block lengths have become one of the most promising candidates for a CV-QKD system (actually, the performance of LDPC codes for a DV-QKD system was also investigated^3^). Herein, multi-edge-type (MET) LDPC codes have attracted much attention due to their excellent performance^17–19^.

As is well-known, the secret key rate is one of the most important performance indices for a QKD system and increasing the repetition rate is one of main methods to increase the secret key rate of a CV-QKD system. Recently, the repetition rate has grown steadily with the experimental progress in this field^5–8,11,12,20^, from MHz to GHz. Correspondingly, high-speed post-processing is required in order to match the high repetition rate. However, one of the bottlenecks that restrict the speed of post-processing for an LDPC-coded CV-QKD system is the throughput of the error correction decoder in the post-processing. To speed up the error correction decoder, several works have been proposed. For example, with the use of a graphics processing unit (GPU), the speed may increase from 7.1 Mb/s^18^, 9.17 Mb/s^17^ to 30.39 Mb/s^19^. However, the speed does not match the growing repetition rate of CV-QKD systems. The key limitation that causes slow decoding speed is due to the fact that successful decoding at very low SNR requires a large number of iterations. In this paper, a layered belief propagation (BP) algorithm^21^ is utilized to speed up the decoding convergence.

In Ref.^22^, the authors proposed a column layered (CL) min-sum decoder to decode the QC-LDPC codes for WiFi (802.11n) and WiMAX (802.16e) and achieved a maximum throughput of 710 Mbits/s. In Ref.^23^, the flooding-based decoder achieve 4.77 Gbits/s with SNR = 5.5 dB, which was implemented on GPU GeForce GTX 1080 Ti by incorporating early termination. This decoder assigns threads to check nodes (CNs) sequentially in the two kernel functions corresponding to CN to variable node (VN) and VN to CN message-passing, and uses atomic operations to complete the synchronization.

In this work, when implementing the GPU-based layered BP decoder, we optimize the storage of the matrix message by merging bits into one number and combine two processes into one kernel to complete a whole iteration. As a consequence, the computation amounts are reduced. We also merge the unrelated sub-matrices because they do not affect each other and can thus be computed simultaneously by threads. The speed of our layered decoder is up to 64.11 Mbits/s for a code of length ${10}^{{6}}$ with a rate of 0.1 under the condition of SNR = 0.161, and 50 iterations without early termination.

## Results

### Implementation of the layered BP decoding algorithm

Given that the messages can be updated at variable/check nodes and can be performed in parallel, the layered BP decoding algorithm is deployed on GPU. This section optimizes the GPU implementation of the layered BP decoding algorithm.

**Figure 1:**
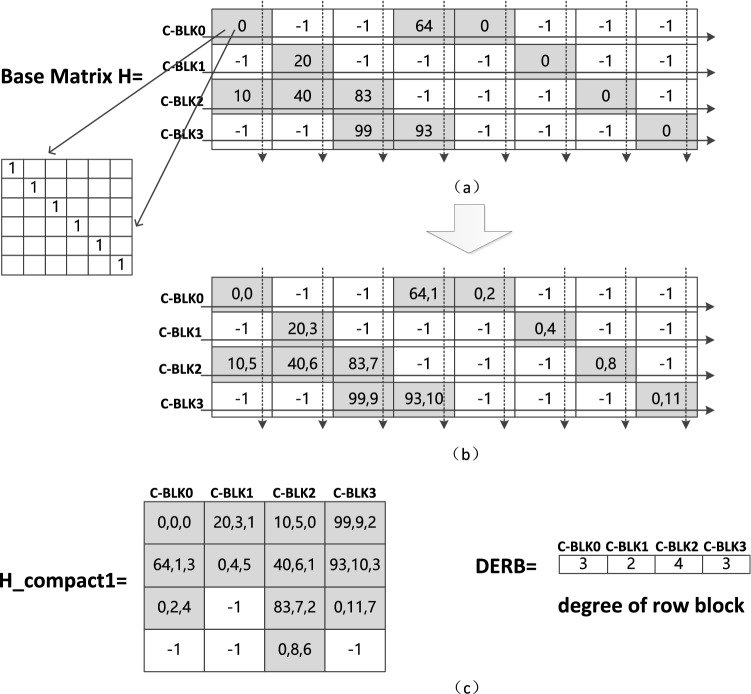
A $4 \times 8$ base matrix and the corresponding file.

The decoder implementation is optimized in such a way that the message is stored in global memory for coalesced access. For memory access in a warp, coalesced access means that the data address of a thread always keeps the same as the thread index, instead of the unordered access. Since the GPU kernel is executed by a warp consisting of 32 threads, the decoding latency can be hidden well for a code with length being a multiple of 32. The layered BP decoder has a coalesced global memory access and stores the parity-check matrix in one file for indexing the corresponding messages. Such a file denoted by H_compact1, will be applied in calculating the messages related to the check nodes. Each element in file H_compact1 contains three pieces of information: the amount of the shift, the position of the element after row rearrangement in the base matrix and the position of the column where the non-negative element located in the base matrix. For example, Fig. 1 displays a 4-by-8 base matrix with an expansion factor of 100. Each non-negative element of the base matrix **H** in Fig. 1a indicates the amount of shift and ‘−1’ represents an all-zero matrix. The second information indicating the position of the element after row rearrangement is demonstrated in Fig. 1b. Then, one sub-matrices shown in Fig. 1c are used for indexing the needed messages. Accordingly, the one-dimensional matrix on the right side in Fig. 1c represent the degrees of the base matrix (i.e., each element of the one-dimensional matrix represents the number of elements that are not ‘-1’ in the corresponding column of the base matrix).

**Figure 2:**
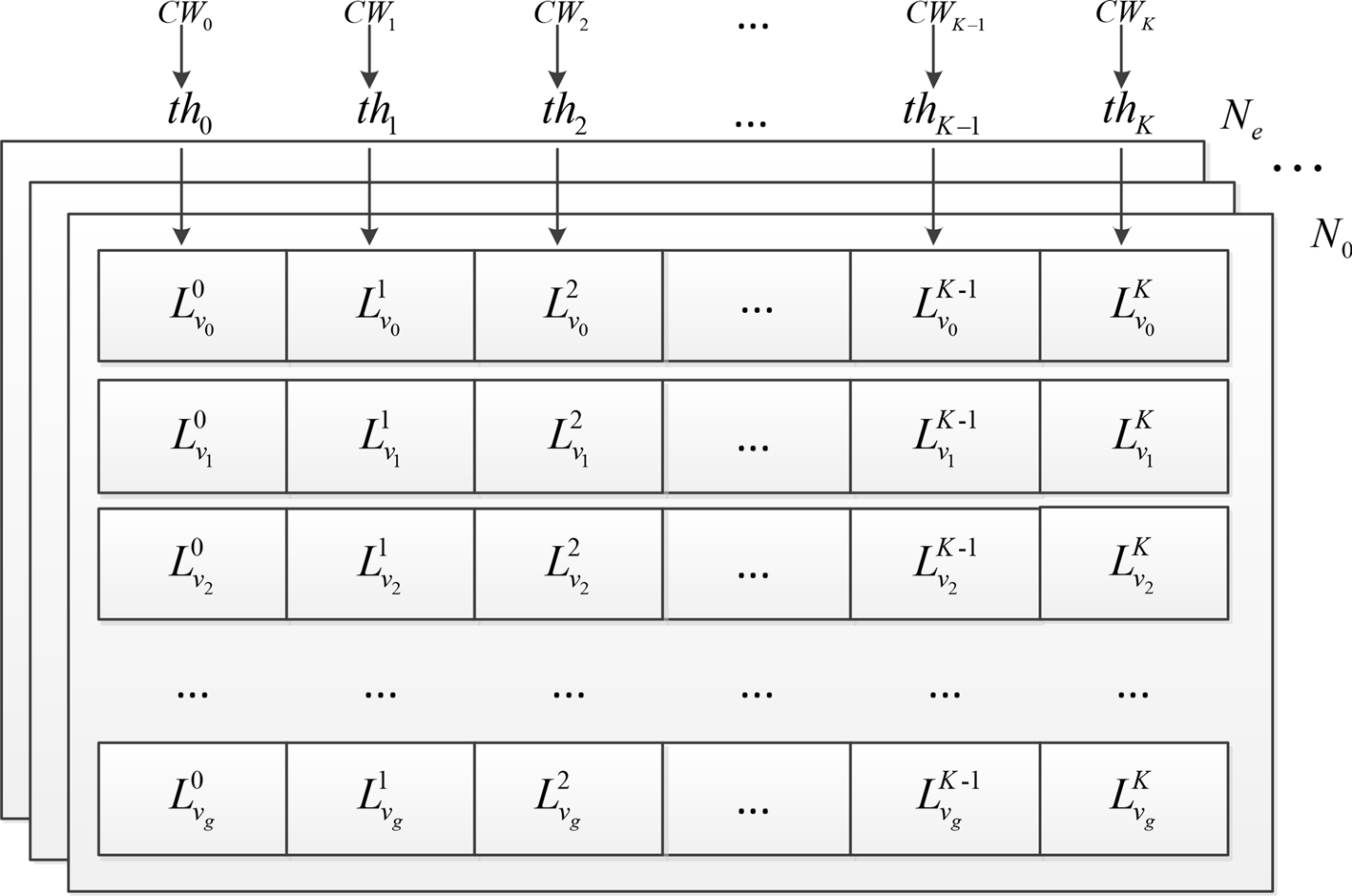
The coalesced access to the message of variable node. Therein, $CW_{0} ...CW_{K}$ represent different codewords, $K + 1$ is the number of codewords decoded at the same time, $N_{0} ...N_{e}$ represent $e + 1$ check nodes, $L_{{v_{i} }}^{k}$ is the message of the *i*-th variable node in the *k*-th codeword, $0 \le i \le g$, $g + 1$ is the total number of variable nodes, and $th_{k}$ is the *k*-th thread, $0 \le k \le K$.

In our decoder, only one kernel is required to complete the iterative process. In this single kernel, one thread maps a kind of information in one codeword, and the same kind of information of all codewords is then stored sequentially. There are three kinds of information, which include: the log-likelihood ratios (LLRs) of variable nodes, the message of check nodes to variable nodes, and the message of variable nodes to check nodes. We store the same kind of information of different codewords sequentially, e.g., $L_{{v_{0} }}^{0}$, $L_{{v_{0} }}^{1}$,…, $L_{{v_{0} }}^{K}$; $L_{{v_{1} }}^{0}$, $L_{{v_{1} }}^{1}$,…, $L_{{v_{1} }}^{K}$ where $L_{{v_{i} }}^{k}$ represents the LLR of the VN $v_{i}$ of the *k*-th codeword denoted by $CW_{k}$. The coalesced access to the message of variable nodes is illustrated in Fig. 2. Therein, the threads $\left( {th_{0} ,th_{1} ,...,th_{K} } \right)$ first map the LLR of $v_{0}$ in codewords $\left( {CW_{0} ,CW_{1} ,...,CW_{K} } \right)$ one by one. Once the LLR of $v_{0}$ is stored, then the thread group maps the LLR of $v_{1}$ in codewords $\left( {CW_{0} ,CW_{1} ,...,CW_{K} } \right)$ until the LLRs of all variable nodes are stored. The message of check nodes to variable nodes or variable nodes to check nodes is also stored in this manner.

**Figure 3:**
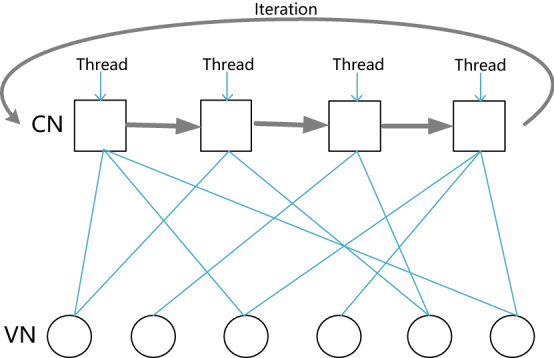
A node parallel decoding scheme in the layered BP decoder.

The GPU-based layered BP decoder updates the messages of variable nodes and check nodes simultaneously, while enabling multiple codewords to be decoded in parallel. For each individual codeword, the required number of GPU threads is the same as the number of check nodes in a sub-matrix. Each thread computes the messages received from the neighboring variable nodes while also calculating the LLR messages for each adjacent variable node. This procedure is illustrated in Fig. 3 by taking an LDPC code with 4 check nodes and 6 variable nodes as an example. Note that, at each iteration, one thread corresponds to a check node. If the expansion factor Z is equal to 100, $1 \times 100$ threads corresponding to check nodes of a sub-matrix send messages to neighboring variable nodes and also calculate the messages from variable nodes. Next the $1 \times 100$ threads are reused to update messages at the second group of check nodes and their neighboring variable nodes. The number of threads that are reused is equal to that of rows of the base matrix. Nonetheless, the layered BP decoder consumes less thread resources and the number of threads assigned to each sub-matrix is only $1 \times 64 \times Z$ (recall that Z is the expansion factor) when decoding 64 codewords simultaneously. The greater the values of Z and the number of codewords are, the higher the utilization rate of the thread is.

**Figure 4:**
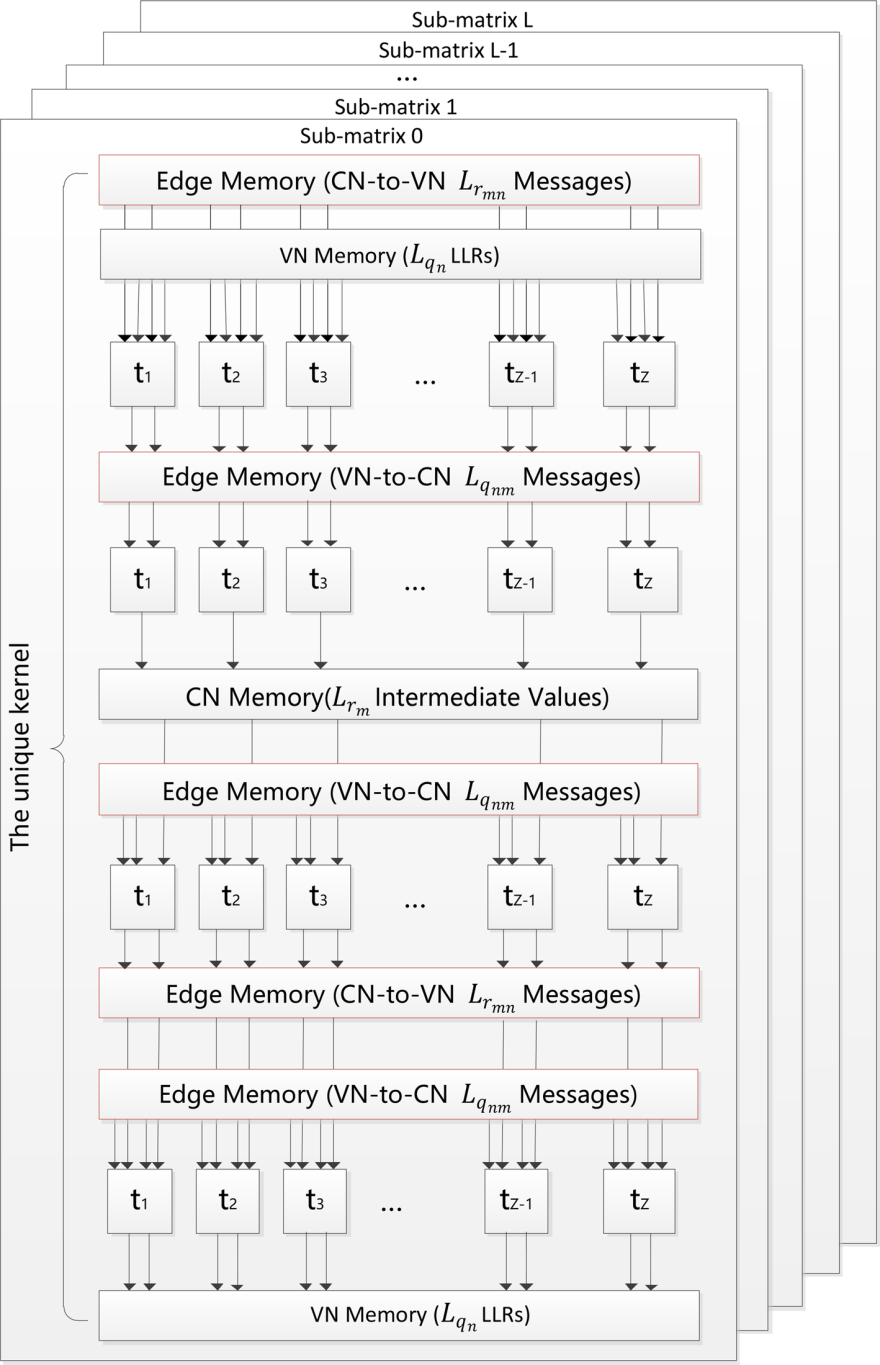
The GPU implementation of the layered decoder showing one multithreaded computation kernel and data flow from top to bottom for one decoding iteration.

The layered BP decoder only involves one file H_compact1 for indexing. This leads to one GPU kernel implementation of the layered BP decoder for each decoding iteration, whose structure is demonstrated in Fig. 4 (This figure is derived from Fig. 4.8 of the thesis^24^). In the unique kernel, the amount of computation in one thread to calculate the message from a variable node to a check node or from a check node to a variable node, defined by the number of edges on which messages are computed, is equal to the degree of the corresponding check node. The message $L_{{q_{nm} }}^{{\left( {t,l} \right)}}$ is updated through $L_{{r_{mn} }}^{{\left( {t - 1,l} \right)}}$ and $L_{{q_{n} }}^{{\left( {t,l - 1} \right)}}$, which represents the message from the *n*-th variable node to the *m*-th check node in the *t*-th iteration and the *l*-th layer. Then each thread calculates the intermediate values $L_{{r_{m} }}^{{\left( {t,l} \right)}}$. In the remaining part of the kernel, each thread computes the message from the *m*-th check node to the *n*-th variable node, denoted by $L_{{r_{mn} }}^{{\left( {t,l} \right)}}$, through $L_{{r_{m} }}^{{\left( {t,l} \right)}}$ and $L_{{q_{nm} }}^{{\left( {t,l} \right)}}$. Afterwards the LLR $L_{{q_{n} }}^{{\left( {t,l} \right)}}$ is updated through $L_{{r_{mn} }}^{{\left( {t,l} \right)}}$ and $L_{{q_{nm} }}^{{\left( {t,l} \right)}}$. There are Z threads in total that are performed at the same time. The decoder accesses message readily by using H_compact1 and the LLRs of variable nodes are delivered to the next layer, i.e., the (*l* + 1)-th layer. The above process is a complete iteration.

The original layered decoder decomposes the **H** matrix into multiple sub-matrices on the basis of layers, which is equivalent to treating each layer as a sub-code. Each sub-matrix utilizes 1 × *Z* threads and the serial calculation is conducted among the sub-matrices. In order to increase the layered decoder’s thread utilization, we merge the unrelated sub-matrices into a new sub-matrix. For example, a 3-by-3 base matrix shown in Eq. (1) with the expansion factor Z can be divided into three sub-matrices and the degree of any variable node in each sub-matrix is equal to one or zero. Herein, a non-negative integer $a$ in Eq. (1) such as ‘1’, ‘0’ and ‘2’ corresponds to a matrix obtained by cyclically shifting the *Z* × *Z* identity matrix to the right by $a$ bits and ‘-1′ indicates the all-zero matrix of *Z* × *Z*.

1 $$ {\mathbf{H}} = \left[ {\begin{array}{*{20}c} 1 & 0 & { - 1} \\ 2 & 1 & 1 \\ 0 & 2 & 0 \\ \end{array} } \right] $$

If a base matrix is of the form given in Eq. (2), we can combine its first two rows into one layer. In other words, the first two rows form a sub-matrix in which the degree of any variable node is equal to one or zero and the third row separately forms a sub-matrix. Two sub-matrices wok in a serial manner by using $2 \times Z$ and $1 \times Z$ threads, respectively.

2 $$ {\mathbf{H}} = \left[ {\begin{array}{*{20}c} 1 & { - 1} & { - 1} \\ { - 1} & 2 & 1 \\ 2 & 0 & 0 \\ \end{array} } \right] $$

Given a base matrix of the form shown in Eq. (3), the first and the third rows of this matrix can be combined into one sub-matrix and the second row forms a sub-matrix.

3 $$ {\mathbf{H}} = \left[ {\begin{array}{*{20}c} 1 & { - 1} & { - 1} \\ 2 & 0 & 0 \\ { - 1} & 2 & 1 \\ \end{array} } \right] $$

The thread utilization rate $\eta$ is computed by

4 $$ \eta = \frac{{T_{1} \times T_{2} \times Z}}{{T_{3} }} $$

where $T_{1}$ is the number of layers in each sub-matrix, $T_{2}$ is the number of codewords, and $T_{3}$ is the total number of threads.

There will be much time needed for the system to call external functions as it is done frequently in a kernel function when using CUDA. Moreover, there will also be some additional waiting time as the warp divergence increases the waiting time when warp threads encounter control flow statements and enter different branches, which means that the remaining branches are currently blocked except for the branch being executed. In this work, the kernel function distinguishes the sign of the input data by calling the application programming interface (API) provided by CUDA, thereby avoiding warp divergence and reducing the calls of external functions. An infinite value or invalid value may appear because of the iterative running of the kernel function. To avoid this, a clipping function included in the CUDA Math API, i.e., device float fminf(), is utilized. With the aid of clipping, the decoding throughput is increased from 60.29 to 64.11 Mbit/s. Another optimization that is being performed is directed to reducing the branches since the branch structure has great drawbacks, especially when different threads utilize different branches with a high probability. For instance, each thread has different calculation amounts and computation time and thus the finished threads need to wait for other unfinished ones. Based on this, we can transform the branch structure to an arithmetic operation when parity checks are used and thereby reduce the decoding time.

### Performance of the proposed GPU-based decoder

**Figure 5:**
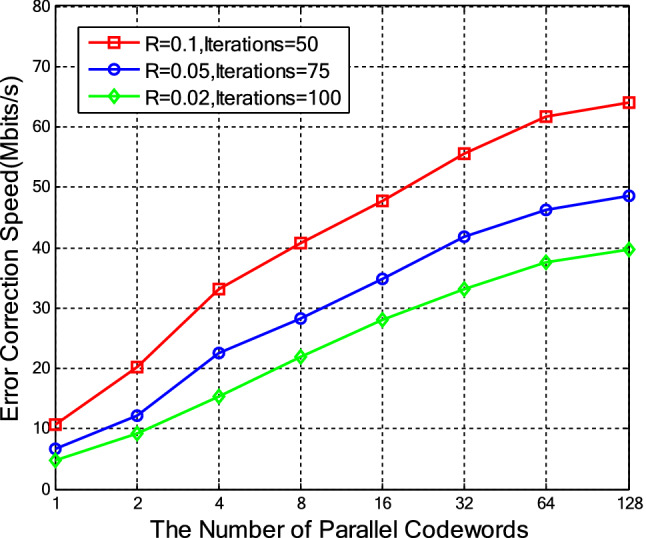
Error correction speed comparison among the different number of decoded codewords in the layered decoder.

The performance of the GPU-based layered BP decoders is investigated for QC-MET-LDPC codes with rates 0.1,0.05 and 0.02 on an NVIDIA TITAN Xp GPU, where the expansion factor is 2,500. The codeword constructed by a cycle elimination algorithm is applied in our work^25^. Figure 5 demonstrates the error correction speed, when different number of codewords are decoded simultaneously. The speed grows steadily from 1 to 128 codewords and it does not converge even if the number of codewords reaches 128. Note that, due to the shortage of storage space, the decoding speed is not considered when the number of codewords decoded simultaneously exceeds 128. Thus, the proposed layered BP decoder in this paper decodes 128 codewords simultaneously and its thread utilization rate is computed by $1 \times 128 \times 2500 \div 67108864 = 0.00477$, where each sub-matrix consists of one layer of the base matrix and the maximum number of threads for NVIDIA TITIAN Xp GPU is 67,108,864.

Table 1 compares the performance of the layered BP decoder with two types of sub-matrices. One type consists of a single layer and the other consists of multiple layers. When decoding 128 codewords of length $10^{6}$ simultaneously, the layered BP decoder with sub-matrices formed by multiple layers performs better than its counterpart in terms of decoding throughput. The improvement is 3.2 Mbits/s, 1.9 Mbits/s and 1.6 Mbits/s, respectively, when three code rates 0.1, 0.05 and 0.02 are considered. Based on this, it appears that combining uncorrelated sub-matrices could be further improved that would then speed up the decoding while also promoting thread utilization.

**Table 1:** Performance comparison of the layered BP decoder when decoding different forms of sub-matrices.

Code rate	0.1	0.1	0.05	0.05	0.02	0.02
SNR	0.161	0.161	0.076	0.076	0.03	0.03
Number of iterations	50	50	75	75	100	100
Form of sub-matrix	Single	Multiple	Single	Multiple	Single	Multiple
Latency per iteration (ms)	42.029	39.931	36.530	35.081	33.720	32.397
Error correction speed (Mbits/s)	60.91	64.11	46.72	48.65	37.96	39.51

## Discussion

As described in Ref.^17^, the early termination scheme can be used as an efficient way to reduce the complexity of LDPC decoder, which avoids unnecessary iterations at high SNR. However, this scheme may not be efficient at low SNR since the decoding often fails after multiple iterations. Table 2 illustrates the performance comparison between the flooding and the layered BP decoders without early termination, when three code rates 0.1 0.05 and 0.02 are considered, respectively. The former, as illustrated in Table 1, can simultaneously decode 64 codewords of length $10^{6}$ while the latter, as illustrated in Table, can simultaneously decode 128 codewords. In the decoding process, the flooding decoder uses the whole matrix whereas the layered BP decoder uses the sub-matrices which consists of unrelated layers of the base matrix. In Table 2, the first row represents code rates and the second row represents is SNR under the BIAWGNC. The third row $\beta$ indicates the reconciliation efficiency related to the code rate and the number of discarded parity bits, which has an influence on the reconciliation distance and the secret key rate. The sixth row represents the number of edges of Tanner graph involved in the decoding and the ninth row represents the average latency of one decoding iteration. The raw throughput $K_{raw}$ in the last row is given by

5 $$ K_{raw} = \frac{{N_{c} \times L_{c} }}{{T_{p} \times T}}\left( {bits/s} \right), $$

**Table 2:** Performance comparison between the flooding and the layered BP decoders with GPU implementation.

Code rate	0.1	0.1	0.05	0.05	0.02	0.02
SNR	0.161	0.161	0.076	0.076	0.03	0.03
$\beta$	92.86%	92.86%	94.63%	94.63%	93.80%	93.80%
Number of iterations	100	50	150	75	200	100
Decoding method	flooding	layered	flooding	layered	flooding	layered
Total number of edges	3,767,500	3,767,500	3,480,000	3,480,000	3,337,500	3,337,500
FER	0.173	0.179688	0.253906	0.25	0.324219	0.328125
Latency per iteration (ms)	42.216	39.931	37.708	35.081	35.635	32.397
Error correction speed (Mbits/s)	30.32	64.11	22.63	48.65	17.96	39.51

where

6 $$ T_{p} = \frac{{T_{all} }}{T}\left( s \right). $$

Herein, *N*_*c*_ is the number of codewords that are decoded simultaneously, $L_{c}$ is the code length, $T_{p}$ is the latency per iteration, and $T$ is the number of iterations. The total decoding latency $T_{all}$ consists of the latency of initialization, iterative decoding, hard decision and memory copy between CPU and GPU. It can be observed that the required number of decoding iterations for the layered decoder is half of that for the flooding decoder. Accordingly, the decoding speed of the layered BP decoder is more than twice that of the flooding decoder and the values for three tested codes is 64.11 Mbits/s, 48.65 Mbits/s and 39.51 Mbits/s, respectively.

Table 3 demonstrates the performance comparison of the layered BP decoders with or without early termination at different SNRs, where the code length is 10^6^ and the code rate is 0.1, respectively. When SNR = 0.161 and 0.171, the layered BP decoder without early termination performs a little faster than that with early termination since less calculations are required to determine whether a valid codeword is obtained in the former. Nevertheless, when SNR = 0.181, the layered BP decoder with early termination is better and the corresponding decoding speed is up to 93.49 Mbits/s. This improvement is attributed to the fact that the introduction of the early termination condition reduces the number of iterations significantly at high SNR.

**Table 3:** Performance comparison of the layered BP decoder when decoding 128 codewords with/without early termination.

SNR	0.161	0.161	0.171	0.171	0.181	0.181
Early termination	No	Yes	No	Yes	No	Yes
FER	0.1797	0.1797	0.0273	0.0273	0	0
Max iterations	50	50	50	50	50	50
Average iterations	50	50	50	50	50	30
Latency per iteration(ms)	39.931	41.463	39.931	41.364	39.931	45.638
Error correction speed (Mbits/s)	64.11	61.74	64.11	61.89	64.11	93.49

As we know, the total secret key rate *K*_*t*_ of a CV-QKD system is given by

7 $$ K_{t} = f \cdot \gamma \cdot K_{r} = f \cdot \gamma \cdot \left( {1 - FER} \right)\left( {\beta I - \chi } \right), $$

where *f* is the repetition rate, *γ* is a constant representing the ratio that the part of the repetition rate is utilized to generate secret key (the remaining part of the repetition rate is used for parameter estimation, signal synchronization, parameter monitoring, etc.), *K*_*r*_ is the secret key rate per pulse, $\beta = \frac{R}{C\left( s \right)}$ is the reconciliation efficiency, $I$ is the mutual information between two participants, $\chi$ is the Holevo bound, $R$ is the code rate and $C\left( s \right)$ is the channel’s capacity. Therefore, a better code, which achieves a lower FER for a given SNR or requires a lower SNR (corresponding to a higher *β*) to achieve a given FER, will bring a higher secret key rate under the condition of the same repetition rate. Moreover, supporting a higher repetition rate by improving the decoding throughput may also lead to a higher secret key rate despite a little higher FER.

**Figure 6:**
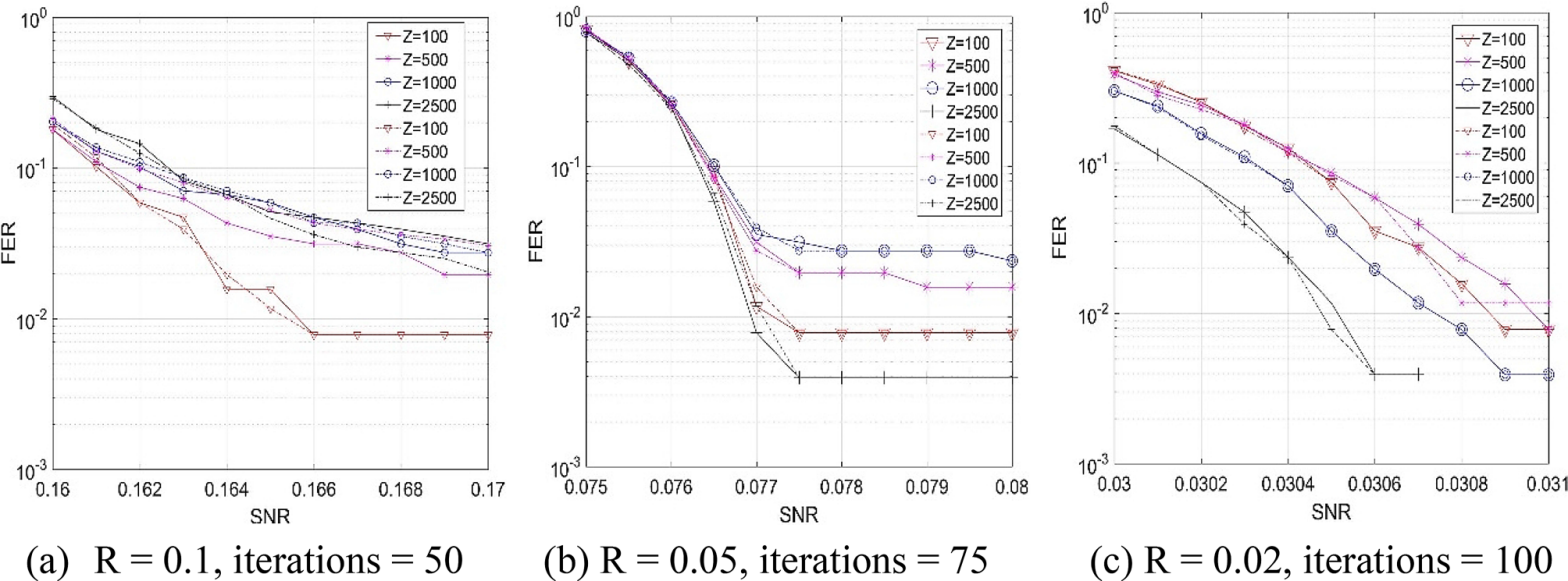
The FER vs SNR curves of the layered (solid lines) and the flooding (dot-dashed lines) decoders.

In our work, we use a large expansion factor since it often brings a large decoding throughput while the FER performance is not necessarily degraded. Figure 6 shows the FER vs SNR curves of the layered and the flooding decoders for three code rates when different expansion factors are considered. From observations of Fig. 6, it can be noted that the codes with Z = 2,500 have the best FER performance among four expansion factors for rates 0.05 and 0.02. Moreover, the layered decoders always have the comparable performance as the flooding decoders for given rates and expansion factors.

We also compare the performance of the layered BP decoder with other decoders described in previous works. As can be seen from Table 4, the author of Ref.^18^ reported a random MET-LDPC code with rate 0.1, where the decoding speed was up to 7.1Mbits/s using a GPU implementation at SNR = 0.161. The decoder was implemented by the flooding BP algorithm without early termination while using internal parallelism and external parallelism. Internal parallelism means several messages are propagated concurrently for a single BP execution corresponding to one message being decoded and external parallelism indicates several vectors are decoded at the same time. In Ref.^17^, the GPU-based decoder decodes a QC-MET-LDPC code with an expansion factor of 512 and achieves 9.17Mbits/s under an early termination condition. Such a decoder consists of four kernels. In the VN to CN message passing kernel, the edge messages are stored in terms of the indices of VNs sequentially, and in the CN to VN message passing kernel, they are stored in terms of the indices of CNs sequentially. Moreover, the authors of Ref.^17^ re-ordered the messages in order to avoid the access to unordered memory.

**Table 4:** Performance comparison with prior works using different types of codes.

Refs. (published year)	Ref.^18^ (2014)	Ref.^17^ (2018)	Ref.^19^ (2018)	Our work
Code type	MET	QC-MET	MET	QC-MET
Code rate	0.1	0.1	0.1	0.1
SNR	0.161	0.161	0.160	0.161
$\beta$	93.10%	92.86%	93.40%	92.86%
Block length	$2^{20}$	$2^{20}$	$10^{6}$	$10^{6}$
Z		512		2,500
Average number of iterations	100	78	100	50
Latency per iteration (ms)	1.477	1.466	21.060	39.931
FER	0.04	0.0243	0.055	0.1797
GPU	AMD Tahiti Graphics Processor	NVIDIA GeForce GTX 1,080	NVIDIA TITAN Xp	NVIDIA TITAN Xp
Error correction speed (Mbits/s)	7.10	9.17	30.39	64.11

Note that if the messages of the degree-1 variable nodes are updated only once at the end of the iterative decoding procedure, the computational complexity and the consumption of the thread will be reduced. As a consequence, the decoding speed of the MET-LDPC code of length $10^{6}$ in Ref.^19^ is up to 30.39 Mbits/s, where the parity check matrix is divided into two files that can then be stored. One stores the CNs adjacent to VNs sequentially in terms of the indices of VNs, the other stores the mapping relations of VNs to CNs. In our work, the proposed layered BP decoder maps the threads to check nodes in each sub-matrix which consists of unrelated rows, and the amount of computation for each thread is in proportion to the degree of a check node. Moreover, the decoder combines three kinds of information related to each nonnegative element of the base matrix into one integer and stores all such integers in a file, which reduces the consumption of GPU memory and the copy time thereby obtaining a high decoding throughput up to 64.11 Mbits/s with no performance degradation.

**Figure 7:**
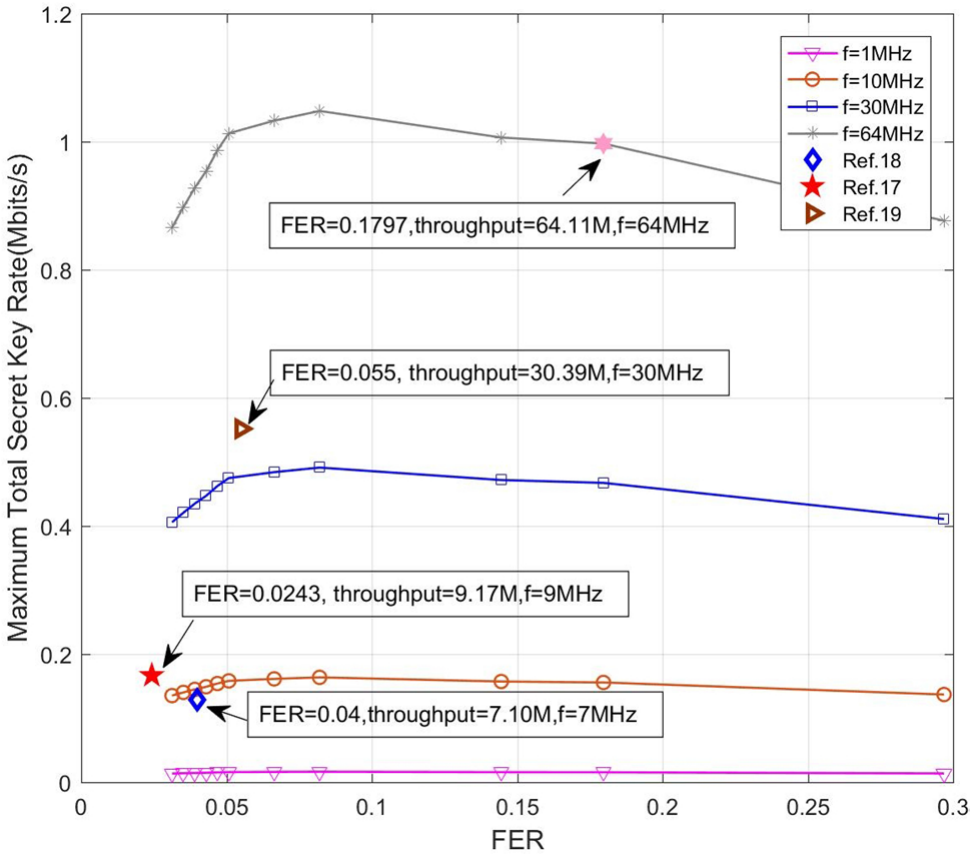
The maximum total secret key rate vs FER curves of the proposed layered decoder with different repetition rates (Four points correspond to FERs and throughputs listed in Table 4).

Figure 7 investigates how the FER relates to the total secret key rate since the key rate is a core index of a QKD system (we assume that the maximum supportable repetition rate is equal to the decoding throughput since the error-correction is usually the most complicated step). Together with the increase of SNR, both FER and *β* decrease. As a consequence, the total secret key rate is not monotonically increasing over the whole FER ranges. One observes that our GPU-based layered decoder outperforms those given in Ref.^17–19^ from the total secret key rate point of view. According to Eq. (7), although our GPU decoder has a higher FER, the improvement on the supportable repetition rate due to the higher decoding throughput is sufficient to compensate the resultant loss of the total secret key rate.

Despite the high throughput of the GPU-based layered BP decoder, there are some shortcomings. For example, most of threads are not used and thus, there exists much space to increase the number of codewords that are decoded simultaneously. In addition, memory shortage may limit the number of parallel decoding. Our future work will focus on reducing memory consumption when decoding one codeword. As a result, thread utilization will then be increased by decoding more codewords simultaneously.

## Methods

In this paper, the three-edge-type QC-LDPC codes are always used^26^. The degree distribution of a MET-LDPC code is specified by a pair of multivariate polynomials *ν*(**r**,**x**) and *μ*(**x**), where *ν*(**r**,**x**) is related to variable nodes and *μ*(**x**) is related to check nodes. The multivariate polynomial pair (*ν*(**r**,**x**), *μ*(**x**)) is defined as follows:

8 $$ \nu ({\mathbf{r}}{,}{\mathbf{x}}) = \sum {\nu_{{{\mathbf{b}}{,}{\mathbf{d}}}} {\mathbf{r}}^{{\mathbf{b}}} {\mathbf{x}}^{{\mathbf{d}}} } , $$

9 $$ \mu ({\mathbf{x}}) = \sum {\mu_{{\mathbf{d}}} {\mathbf{x}}^{{\mathbf{d}}} } , $$

where **b** represents different types of channels (Typically, b only has two values, i.e., 0 or 1, corresponding to two channels which transmit bits and puncture bits, respectively), **d** denotes the degrees of different edge types, **r** represents variables corresponding to the different types of channels, and **x** denotes variables related to edge types. Moreover, *ν*_**b**,**d**_ and *μ*_**d**_ denote the probabilities of variable nodes of type (**b**, **d**) and check nodes of type **d**, and the code rate is computed by

10 $$ R{ = }\sum {\nu_{{{\mathbf{b}}{,}{\mathbf{d}}}} } { - }\sum {\mu_{{\mathbf{d}}} } $$

The construction of MET-QC-LDPC codes used in our simulations is described as follows.

Step 1: Generating the degree distribution of a MET-LDPC code for a given code rate. In this paper, three degree distribution functions corresponding to three rates 0.1, 0.05 and 0.02, respectively, is given below^27^:

- R = 0.1: $$\nu ({\mathbf{r}}{,}{\mathbf{x}}) = 0.0775r_{1} x_{1}^{2} x_{2}^{20} + 0.0475r_{1} x_{1}^{3} x_{2}^{22} + 0.875r_{1} x_{3}$$, $$\mu ({\mathbf{x}}) = 0.0025x_{1}^{11} + 0.0225x_{1}^{12} +$$$$0.03x_{2}^{2} x_{3} + 0.845x_{2}^{3} x_{3}$$
- R = 0.05: $$\nu ({\mathbf{r}}{,}{\mathbf{x}}) = 0.04r_{1} x_{1}^{2} x_{2}^{34} + 0.03r_{1} x_{1}^{3} x_{2}^{34} + 0.93r_{1} x_{3}$$, $$\mu ({\mathbf{x}}) = 0.01x_{1}^{8} + 0.01x_{1}^{9} + 0.41x_{2}^{2} x_{3} { + }$$$$0.52x_{2}^{3} x_{3}$$
- R = 0.02: $$\nu ({\mathbf{r}}{,}{\mathbf{x}}) = 0.0225r_{1} x_{1}^{2} x_{2}^{57} + 0.0175r_{1} x_{1}^{3} x_{2}^{57} + 0.96r_{1} x_{3}$$, $$\mu ({\mathbf{x}}) = 0.010625x_{1}^{3} + 0.009375x_{1}^{7}$$$${ + }0.6x_{2}^{2} x_{3} + 0.36x_{2}^{3} x_{3}$$

Step 2: According to the degree distribution, construct the base matrices by using the progressive-edge-growth (PEG) algorithm^28^ and make its girth as large as possible.

Step 3: Extend the base matrix with circulant permutation matrices, where ‘0’ elements are replaced by *q* × *q* zero matrices and ‘1’ elements are replaced by *q* × *q* cyclically-shifted identity matrices with randomly generated cyclic shifts.
